# Effect of Non-pharmaceutical Interventions on COVID-19 in Rwanda: An Observational Study

**DOI:** 10.1007/s44197-023-00094-4

**Published:** 2023-03-09

**Authors:** Edson Rwagasore, Olivier Nsekuye, Alfred Rutagengwa, Ziad El-Khatib

**Affiliations:** 1 Emergency Preparedness and Response Division (PHS&EPR), Public Health Surveillance, Rwanda Biomedical Center (RBC), PO Box 7162, Kigali, Rwanda; 2 World Health Organization Rwanda Country Office, PO Box 1324, Kigali, Rwanda; 3 Department of Global Public Health, Karolinska Institutet, 17177 Solna, Sweden; 4 Bill and Joyce Cumming Institute of Global Health, University of Global Health Equity, Kigali, 6955 Rwanda

**Keywords:** Rwanda, Non-pharmaceutical interventions, COVID-19, Global health

## Abstract

**Background:**

On 11 March 2020, COVID-19 was declared as a pandemic by the World Health Organization (WHO). The first case was identified in Rwanda on 24 March 2020. Three waves of COVID-19 outbreak have been observed since the identification of the first case in Rwanda. During the COVID-19 epidemic, the country of Rwanda has implemented many Non-Pharmaceutical Interventions (NPIs) that appear to be effective. However, a study was needed to investigate the effect of non-pharmaceutical interventions applied in Rwanda to guide ongoing and future responses to epidemics of this emerging disease across the World.

**Methods:**

A quantitative observational study was conducted by conducting analysis of COVID-19 cases reported daily in Rwanda from 24 March 2020 to 21 November 2021. Data used were obtained from the official Twitter account of Ministry Health and the website of Rwanda Biomedical Center. Frequencies of COVID-19 cases and incidence rates were calculated, and to determine the effect of non-pharmaceutical interventions on changes in COVID-19 cases an interrupted time series analysis was used.

**Results:**

Rwanda has experienced three waves of COVID-19 outbreak from March 2020 to November 2021. The major NPIs applied in Rwanda included lockdowns, movement restriction among districts and Kigali City, and curfews. Of 100,217 COVID-19 confirmed cases as of 21 November 2021, the majority were female 51,671 (52%) and 25,713 (26%) were in the age group of 30–39, and 1866 (1%) were imported cases. The case fatality rate was high among men (*n* = 724/48,546; 1.5%), age > 80 (*n* = 309/1866; 17%) and local cases (*n* = 1340/98,846; 1.4%). The interrupted time series analysis revealed that during the first wave NPIs decreased the number of COVID-19 cases by 64 cases per week. NPIs applied in the second wave decreased COVID-19 cases by 103 per week after implementation, while in the third wave after NPIs implementation, a significant decrease of 459 cases per week was observed.

**Conclusion:**

The early implementation of lockdown, restriction of movements and putting in place curfews may reduce the transmission of COVID-19 across the country. The NPIs implemented in Rwanda appear to be effectively containing the COVID-19 outbreak. Additionally, setting up the NPIs early is important to prevent further spread of the virus.

## Introduction

In January 2020, the World Health Organization (WHO) declared the novel Coronavirus (COVID-19) as a public health emergency outbreak of international concern. Worldwide, different containment measures and interventions were implemented to slow transmission [1]. However, COVID-19 continued to spread globally and continues to present unprecedented challenges worldwide. By the 21st November 2021, a total of 256,710,541 confirmed cases and 5,154,523 deaths had been reported globally [2]. Rwanda confirmed its first case of COVID-19 on 14th March 2020. As of the 21st November 2021, the Rwanda Biomedical Center (RBC) confirmed a total of 100,217 cases and 1340 deaths related to COVID-19 [3]. Many countries implemented a series of non-pharmaceutical interventions (NPIs), such as isolating COVID-19 confirmed cases, contact tracing, quarantine of exposed individuals, travel restrictions, school and workplace closures, cancellation of mass gatherings, handwashing or using hand sanitizers, total lockdown, border restrictions and use of masks [4–6]. Considering the quick spread of COVID-19 worldwide, and as there were no pharmaceutical interventions to treat this novel infectious disease, NPIs were among the few tactics to deal with the pandemic. By November 2021, Rwanda experienced three different waves of COVID-19. Apart from NPIs such as wearing masks indoors and outdoors, social distancing, handwashing or using hand sanitizers, mandatory isolation after a confirmed case, contact tracing, a mandatory quarantine of exposed individuals for 14 days, and testing, there were also lockdowns, movement restrictions between districts, and curfews used to flatten the curve during the three waves experienced [7, 8]**.** Different studies assessing the effects of NPIs were conducted in different countries, and they showed that NPIs have a tremendous role in reducing the spreading of COVID-19 in different settings [9–13]. The NPIs applied in Rwanda indicated an effect on the reduction of COVID-19 cases during different waves recorded. However, limited studies have been conducted in Rwanda on the effect of NPIs to contain the spread and reduce the size of the COVID-19 outbreak. In this study, we assessed the effect of three NPIs (lockdowns, population movement restriction, and curfews) on reducing COVID-19 transmission in Rwanda.

### Description of Major Non-pharmaceutical Interventions Over Time in Rwanda

After the first case of COVID-19, on 14th March 2020, the government of Rwanda implemented different preventive measures (e.g., the closure of schools, religious places, nightclubs, restriction of unnecessary movements in the country and on all borders, and the use of the 114 toll-free number to report suspected COVID-19 symptoms).

On 22nd March 2020 to 4 May 2020, Rwanda implemented a total lockdown measure of the whole country. After easing the total lockdown, the country conducted regular mass testing countrywide on a monthly basis. Curfews and population movement restrictions measures have also been used to reduce transmission in some districts of the country based on the data obtained from conducted mass testing. Along with these interventions, there were several measures that were used continuously in the general population without considering the existing number of new cases since the confirmation of COVID-19 pandemic in Rwanda. Those NPIs included wearing masks in public places, practice of social distancing and hand washing. These measures were reinforced through different channels of communication, local leaders and community engagement.

**Fig. 1:**
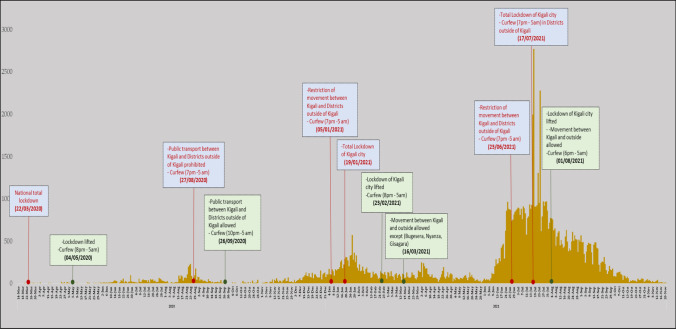
Daily COVID-19 confirmed cases with major Non-pharmaceutical interventions (NPIs) applied in Rwanda over time

*The first wave* was declared in the middle of August 2020, when COVID-19 transmission reached the community level (Fig. 1).

During 27 August 2020 through 26 September 2020, there was a restriction of the movement from/to the capital city Kigali, in addition of curfews between 7.00 pm until 5.00 am. This measure was lifted when a low number of cases was recorded. However, curfews continued to be implemented by adjusting curfew hours based on the trend of new cases.

*The second wave* of new COVID-19 cases occurred from the end of November 2020 and 5th January 2021. A rise in the number of COVID-19 cases was observed and the government started tightening existing measures to contain the spread of the virus among the community.

*On 5th January 2021*, in addition to the existing measures, public and private transportation to/from Kigali city, and between different districts was prohibited, and curfews between 7.00 pm until 5.00 am were implemented.

On 19th January 2021, a full lockdown in the city of Kigali was made due to the continuous increase of cases.

*On 23rd February 2021*, lockdown in Kigali city was eased as new COVID-19 cases decreased. The existing measures were maintained and curfews started at 8.00 pm through 5.00 am.

*On 16th March 2021*, movements to/from Kigali city to other districts were allowed except to the three districts (Bugesera, Gisagara and Nyanza) that were still reporting a high number of new cases.

*The third wave was announced at the end of June 2020*, when another sharp increase of new cases was observed, and many cases were being recorded in the city of Kigali, mostly driven by the Delta variant [14].

*On 23rd June 2021*, the government of Rwanda implemented the restriction of movement from/to the city of Kigali, along with a curfew starting at 7.00 pm to 5.00 am.

*On 17th July 2021*, the city of Kigali was put under a full lockdown with a curfew implemented from 7.00 pm until 5.00 am in other districts.

*On the 1st August 2021*, the lockdown of the city of Kigali was ended and movements to/from Kigali city were allowed. However, curfews were applied at 6.00 pm through 5.00 am.

## Methods

### Study Design and Setting

This was a quantitative observational study that included all COVID-19 cases reported across Rwanda as of November 21, 2021. Rwanda is located in Central Africa, and it is subdivided into five provinces (Northern Province, Southern Province, Eastern Province, Western Province and Kigali city). These provinces are further split into 30 districts. Districts are further split into 416 sectors, sectors split into 2148 cells that are further split into 14,837 villages. The country of Rwanda has 26,338 km^2^ of surface, and a population of 12,955,736 [13, 15]. The start of a COVID-19 wave in Rwanda was defined when a seven day-incidence rate shifted from:

i. Low to either moderate, high, or very high
ii. Its end was considered when a 7-day incidence rate shifts back to low

A 7-day incidence rate categorization was defined as follows:

- Low: ≤ 5 cases/100,000 population
- Moderate: > 5–24 cases/100,000 population
- High: ≥ 25–50 cases/100,000 population
- Very high: > 50 cases/100,000 population

Following these guidelines, Rwanda has recorded three waves of COVID-19, and three major NPIs have been implemented to control, nationally, the COVID-19 transmission (Fig. 1):

1. The first intervention included a *total lockdown* that helped to restrict movement and reduce contacts and gatherings among the general population. National lockdown was implemented on 22nd March 2020 after identification of the first case of COVID-19 in Rwanda. Kigali city, Districts or Sectors were put under lockdown as time evolved based on a high number of cases recorded per area.
2. The second intervention included *the restriction of* population movements between the city of Kigali and the remaining districts of the country, to reduce the transmission of COVID-19 between districts and the City of Kigali.
3. The third intervention included *curfews* for social gatherings (i.e. restaurants, bars and other public events) implemented due to COVID-19 new cases situation. Curfews were starting either at 6 pm, 7 pm, 8 pm, 9 pm or 10 pm evening until 5 am morning.

### Data Source

We conducted data analysis of cases diagnosed with COVID-19 that were reported daily by the Ministry of Health (MoH) from 14 March 2020 to 21 November 2021 to assess the effect of NPI on COVID-19. Data were published daily on the Twitter account of MoH [16] and Rwanda Biomedical Centre (RBC) website [3]. A confirmed case was defined as a person with laboratory confirmation on Reverse Transcription-Polymerase Chain Reaction (RT-PCR) test or on COVID-19 Rapid diagnostic test, irrespective of clinical signs and symptoms [17]. All health facilities countrywide including private clinics have the ability to test COVID-19 with a rapid diagnostic test. PCR test is conducted by National Laboratory Reference and some hospitals that are equipped with PCR machines.

### Dependent and Independent Variables

The primary dependent variable was COVID-19 cases for the data collected daily from the tweets of the Ministry of Health and from the RBC website, while the main independent variable was the NPIs intervention in relation to two different intervention calendar time periods (i.e., before and after each intervention).

### Sampling and Statistical Analysis

This study included the numbers of all COVID-19 cases reported across Rwanda (from all 30 districts) between 14 March 2020 through 21 November 2021.

For the statistical analysis, we reported frequencies of cases and socio-demographic information (sex, age groups and source of transmission). Incidence rates expressed in cases per 100,000 population were calculated by dividing new cases recorded over the general population. The changes in COVID-19 cases before and after NPIs were evaluated by using interrupted time series (ITS) analysis where an outcome variable is observed over multiple, equally-spaced time periods before and after the introduction of an intervention that is expected to interrupt its level or trend. It also estimates the effect of an intervention on an outcome variable either for a single treatment group or when compared with one or more control groups [18]. Data analysis and maps development were done using Stata software version 16 and ArcGIS, respectively.

## Results

### Distribution of COVID-19 Confirmed Cases and Deaths in Rwanda from 14 March 2020 to 21 November 2021

As of 21 November 2021, Rwanda reported 100,217 cases and 1340 deaths. Women had a higher proportion of COVID-19 cases 52% (*n* = 51,671) compared to men. The age group of 30–39 had the highest proportion of 26% (*n* = 25,713) followed by the age group of 20–29 with 24% (*n* = 23,714). A minority of the cases (1%; *n* = 1371) were imported cases from countries outside of Rwanda. Men had a higher case fatality rate (1.5%) compared to women, while the age group of ≥ 80 showed the highest case fatality rate of 16.6% (*n* = 309/1866) compared to other age groups (Table 1).

**Table 1:** Number of COVID-19 cases and deaths recorded from 14 March 2020 to 21 November 2021 in Rwanda

COVID-19 cases			COVID-19 death		
Characteristic	Frequency (*n*)	Proportion (%)	Frequency (*n*)	Proportion (%)	Case fatality rate (%)
Sex	*N* = 100,217		*N* = 1340		1.3
Men	48,546	48	724	54	1.5
Women	51,671	52	616	46	1.2
Age group					
< 20	14,578	15	26	2	0.2
20–29	23,714	24	39	3	0.2
30–39	25,713	26	97	7	0.4
40–49	15,536	16	123	9	0.8
50–59	9379	9	177	13	1.9
60–69	6480	6	292	22	4.5
70–79	2951	3	277	21	9.4
> 80	1866	2	309	23	16.6
Source of transmission					
Local	98,846	99	1340	100	1.4
Imported	1371	1	0	0	0.0

### Description of the Trend of COVID-19 Incidence Rate (IR) in Rwanda from 14 March 2020 to 21 November 2021

**Fig. 2:**
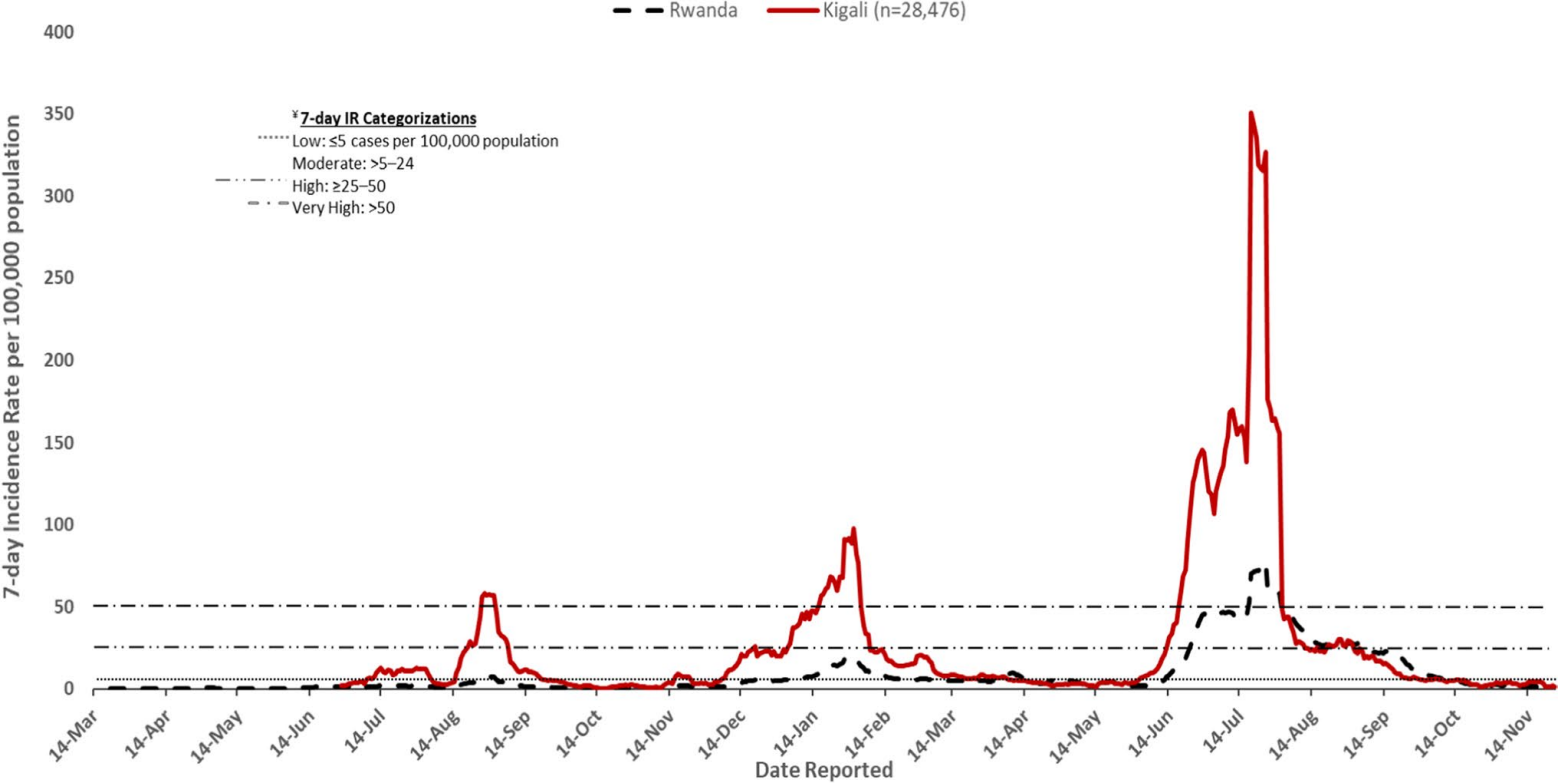
Trend of incidence rate (IR) in the City of Kigali and Rwanda from 14 March 2020 to 21 November 2021

As shown on Fig. 2 below, Rwanda experienced the unexpected increase in incidence rate three times that were deemed as three waves of COVID-19. The first wave was observed in the month of August 2020 where incidence rate in Rwanda increased from low incidence rate (< 5 cases per 100,000 population) to moderate incidence rate (> 5–24 cases per 100,000 population) while in Kigali city increased from low incidence rate (< 5 cases per 100,000 population) to very high incidence rate (> 50 cases per 100,000 population).

The second wave was noticed in the month of January 2021 where incidence rate in Rwanda increased from low incidence rate (< 5 cases per 100,000 population) to moderate incidence rate (> 5–24 cases per 100,000 population) while in Kigali city increased from low incidence rate (< 5 cases per 100,000 population) to very high incidence rate (> 50 cases per 100,000 population).

Then, the third wave was observed in June–July 2021. The incidence rate of Rwanda increased from low incidence rate (< 5 cases per 100,000 population) to very high incidence (> 50 cases per 100,000 population) while in Kigali city it increased from low incidence rate (< 5 cases per 100,000 population) to very high incidence rate (> 50 cases per 100,000 population).

### Comparison of COVID-19 Incidence Rate Before and After the Implementation of Major Non-pharmaceutical Interventions Among Districts of Rwanda

**Fig. 3:**
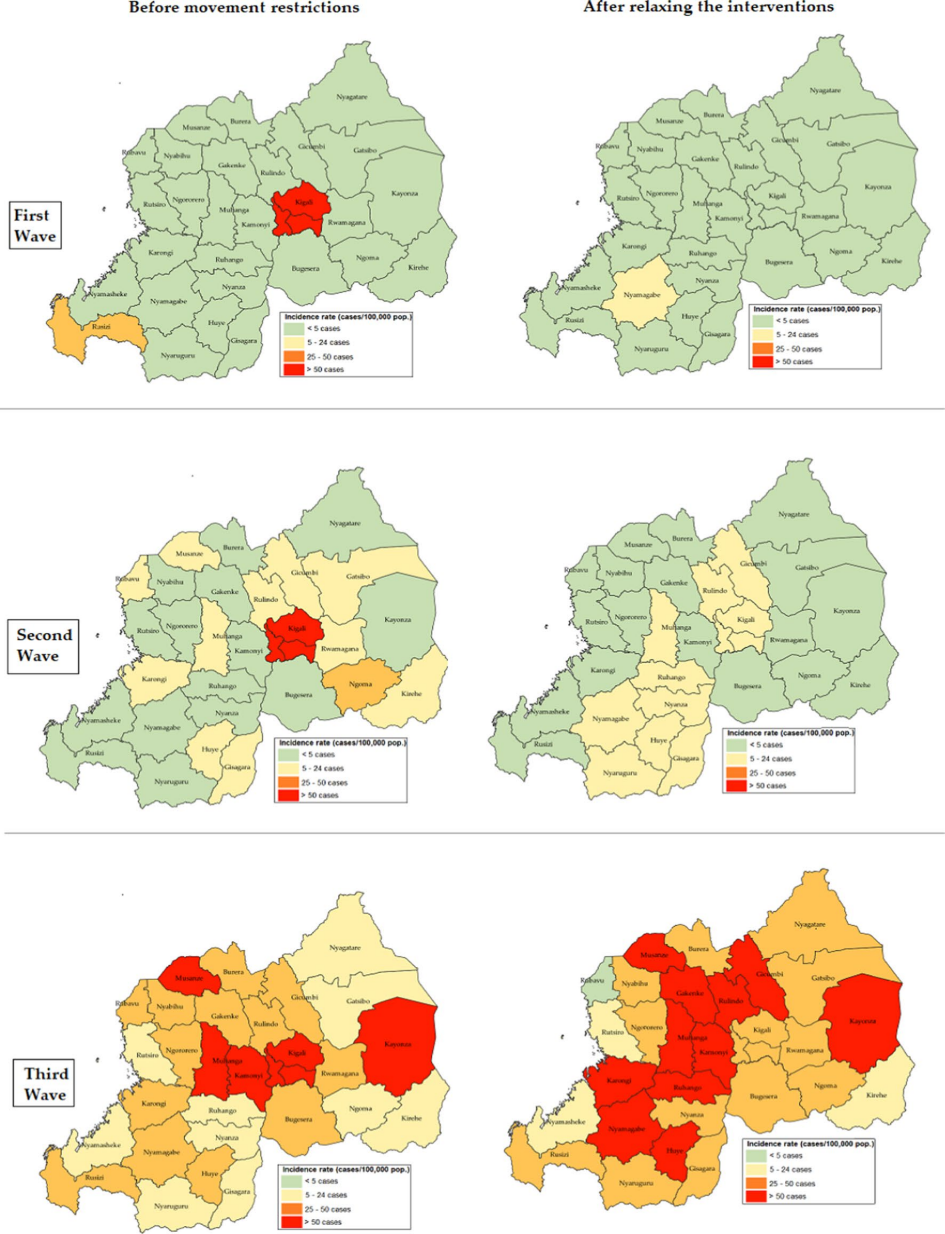
Maps comparing cumulative incidence rates (new cases of COVID-19 since the confirmation of the first case per 100,000 population) by district in Rwanda before and after implementation of major non-pharmaceutical interventions for each wave

Figure 3 shows maps of Rwanda comparing COVID-19 incidence rate by districts before and after implementation of major NPIs in the country for each wave that occurred in Rwanda. During the first wave, before major NPIs implementation in addition to existing measures to contain the spread of the virus in the community, Kigali city was the most affected district of Rwanda. Kigali city had a very high incidence rate (> 50 cases per 100,0000 population), along with a high incidence rate in Rusizi District (> 25–50 cases per 100,000 population). After easing major interventions during the first wave, all districts showed a low incidence rate of < 5 cases per 100,000 people except Nyamagabe district that was still indicating a moderate incidence rate of 5–24 cases per 100,000 people. In the second wave, Kigali city also reported a very high incidence rate (> 50 cases per 100,0000 population) followed by Ngoma District that was experiencing a high incidence rate (> 25–50 cases per 100,000 population) while other districts were presenting either moderate (5–24 cases/100,000 population) or low (< 5 cases/100,000 population) incidence rates. The major NPIs were withdrawn when all districts of Rwanda and the city of Kigali were presenting either moderate (5–24 cases/100,000 population) or low (< 5 cases/100,000 population) incidence rate. During the third wave, as the country was in the phase of endemicity of COVID-19 and mitigating its effects among the community. Before implementing major NPIs, a very high incidence rate (> 50 cases/100,000 population) was observed in Kigali city, which was then reduced to a high incidence rate (25–50 cases/100,000 population) on 1st August 2021 after implementing measures to halt this third wave. However, in the remaining Districts that were still reporting a very high incidence, sectors were put under lockdown to limit the transmission.

### Description of An Interrupted Time Series Analysis (ITSA) to Compare the Trend of Cases Before and After the Implementation of Major NPIs in Rwanda Over Time

**Fig. 4:**
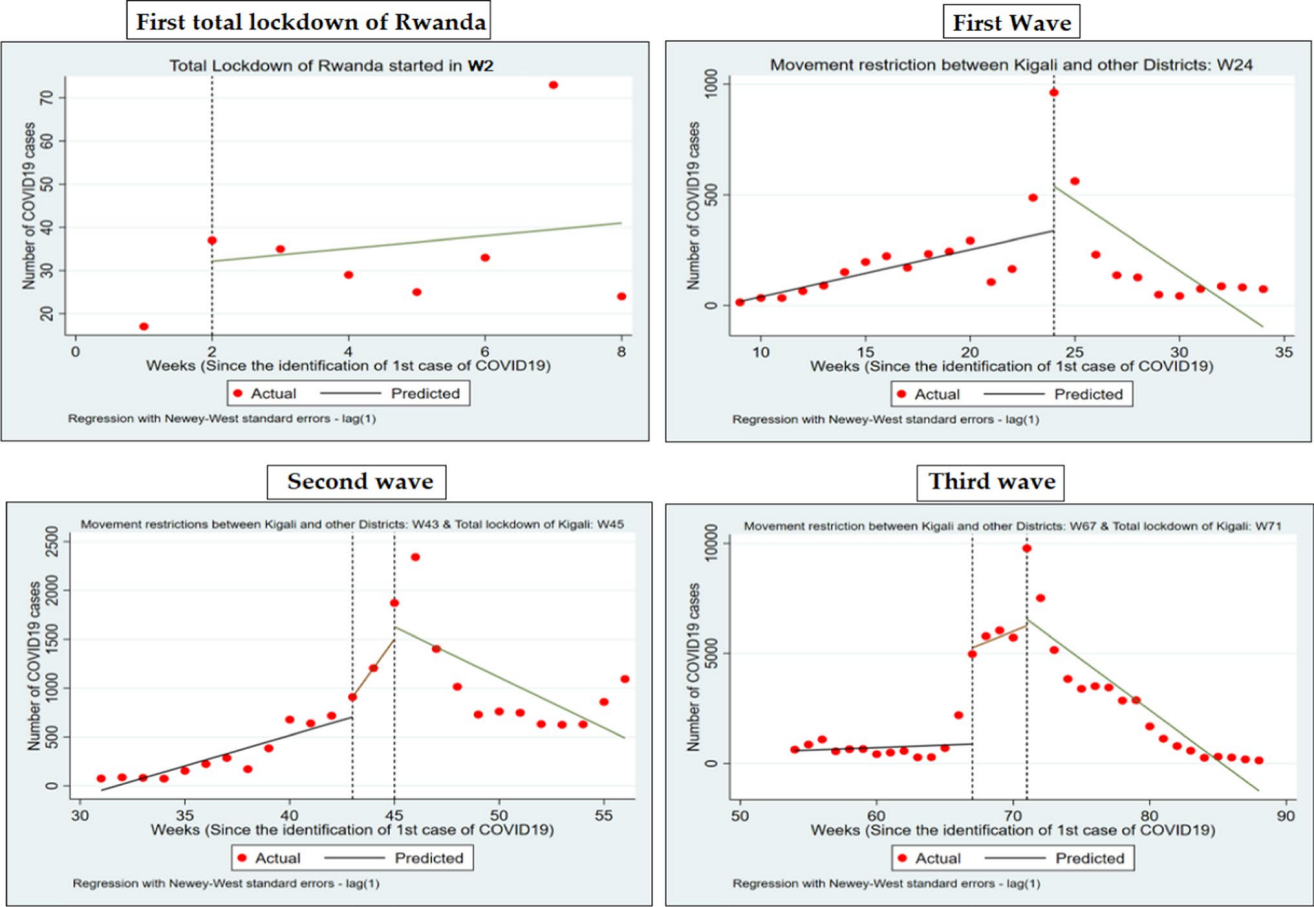
Interrupted time series analysis (ITSA) comparing period before and after major NPIs intervention in Rwanda

As shown in Fig. 4, the total lockdown of Rwanda applied on 22 March 2020 helped to maintain COVID-19 cases at low level. A slight weekly increase in COVID-19 cases after lockdown was observed with a non-significant increase of 2 cases per week (*p* = 0.61) from 22 March 2020 to 04 May 2020. During the first wave observed in July 2020, the major NPI applied was the restriction of movement between Kigali city and other districts of Rwanda combined with a curfew from 7 pm to 5 am. These interventions helped to reduce the number of COVID-19 cases with a significant weekly decrease of 64 cases per week (*p* = 0.02) from 27 August 2020 to 26 September 2020. In January 2021, the second wave was observed. On 05 January 2021, the first major NPIs were movement restriction between Kigali city and other districts of Rwanda combined with curfew from 7 pm to 5 am. However, during these interventions, cases continued to increase significantly, with 297 cases per week (*p* < 0.001) till 19 January 2021. From 19 January 2021 to 23 February 2021, Kigali city was under lockdown. However, movements between Kigali and other districts was allowed on 13 March 2021. Since the application of Kigali city lockdown till the allowance of movements between Kigali and other districts, a weekly significant decrease in number of cases was observed with a weekly decrease of 103 cases per week (*p* < 0.001).

During the third wave observed in June–July 2021, movement between Kigali city and other districts were prohibited on 23 June 2021, alongside a curfew of 7 pm to 5 am. This intervention did not lead to a reduction in cases because a significant weekly increase of 249 cases per week (*p* = 0.03) was noticed. On 17 July 2021, Kigali city was put under total lockdown which ended on 01 August 2021. Movements between Kigali and other districts were also allowed but the curfew was lengthened from 6 pm to 5 am. Since lockdown of Kigali city, a significant weekly decrease of 459 cases per week was observed (*p* < 0.001) (Table 2).

**Table 2:** Summary the estimated slope, confidence interval, and *p* values from ITSA

Linear trend	Coefficient	Standard error	Estimated slope	*p* value	Confidence interval
Total country lockdown	1.464	2.723	0.538	0.614	− 5.536 to 8.465
1st wave	− 63.609	26.894	− 2.365	0.027	− 119.384 to − 7.834
2nd wave	− 103.388	49.630	− 2.083	0.050	− 206.915 to 0.139
3rd wave (1st intervention)	249.4	112.3	2.220	0.030	19.723 to 479.076
3rd wave (2nd intervention)	− 458.667	92.455	− 4.960	0.000	− 647.76 to − 269.57

## Discussion

The implementation of the main NPIs such as lockdown, movement restrictions, and curfews in Rwanda was associated with a decrease in the transmission of COVID-19. The evolution of COVID-19 among Rwandan districts was differently observed due to the changes in movement patterns and behaviors of humans, and so the severity of the COVID-19 outbreak in Rwanda varies from one district to another. However, our results indicated that population movement, contact, and interaction among the population had a major role in the transmission of COVID-19.

Lockdowns were among chosen measures to contain COVID-19 disease from spreading in the population in Rwanda when reported cases were increasing at a very high incidence rate (> 50 cases/100,000 population). In general, Rwanda applied the first total lockdown after identifying the first cases that were mostly imported from outside countries. Other lockdowns were also implemented in different areas like Kigali city where the number of new cases were increasing abundantly. This study indicated that lockdowns are more effective to reduce the number of COVID-19 cases and controlling the spread of disease among population. These findings were similar to an interrupted time-series study in Hubei and Guangdong provinces in China before and after lockdown, which showed a significant reduction in the incidence of cases, indicating the effectiveness of lockdown in containing the outbreak [10]. Other studies conducted in the USA and Hong Kong have also illustrated the effectiveness of applied lockdown to flatten the COVID-19 curve [11, 12].

Movement restriction as one of the major NPIs applied in Rwanda aimed to reduce contact of potential cases with others, and it was effective at slowing the outbreak. This intervention was successful during the first wave in combination with curfews. However, during the second and third waves, it was seconded with lockdown to successfully flatten the curve of new COVID-19 cases. This agreed with the study conducted in the United Kingdom that included various transmission routes and mitigation measures and suggested that movement restrictions alone will not eliminate transmission, and that a combination of stricter measures is required. However, a study conducted in Malaysia indicated that movement restrictions have a synergistic effect on controlling COVID-19 outbreaks [13, 19]. The third major intervention that has been applied alongside the other existing interventions was curfew. This approach targets social interactions among family members, friends or close acquaintances, where social distancing is likely to be more laidback. This was found to be more effective when combined with NPIs including movement restriction in addition to other measures like wearing masks, social distancing, hand hygiene, etc. Curfews were applied since the easing of the first lockdown in Rwanda till 21 November 2021. However, curfew hours have been dynamically changed based on the trend of COVID-19. When cases were increasing, curfews were enforced during early hours. This study indicated that curfews, in combination with other measures that were in place, kept COVID-19 cases at a low level in Rwanda. However, a study conducted in Germany indicated that curfews are unlikely to reduce the absolute number of contacts, as many people would adhere to the rules by meeting earlier, potentially increasing contact density during the day [20].

Our study has strengths of using all data captured on confirmed cases of COVID-19 in Rwanda and assessing the main three interventions that were used by many countries worldwide to lower the spread of COVID-19 infection. These measures can be applied in most countries worldwide to control the outbreak of COVID-19. Our study had a limitation, as we could not adjust for other NPIs such as mask wearing, isolation and quarantine , personal hygiene or other COVID-19 related prevention measures. Although we assumed that all measures were applied in a consistent way throughout the country, we recommend further modelling studies to investigate their roles in containing the outbreak in Rwanda. In addition, the number of observations was limited between movement restrictions and lockdown in the second and third waves.

## Conclusion

Our findings suggest that early implementation of lockdown, restriction of movements and enforcing curfews may reduce the transmission of COVID-19 across Rwanda. Lockdown seems to be more effective compared to other two types of NPIs. The simultaneous implementation of two or more types of NPIs may be the most effective for containing the spread of COVID-19.

## Data Availability

All data and materials are available upon the contact with the authors.
